# Fabrication, Characterization, and Antimicrobial Activity of Carvacrol-Loaded Zein Nanoparticles Using the pH-Driven Method

**DOI:** 10.3390/ijms23169227

**Published:** 2022-08-17

**Authors:** Huaming Zheng, Jiangli Wang, Feng You, Mingyu Zhou, Shengwei Shi

**Affiliations:** Hubei Key Lab of Plasma Chemistry and Advanced Materials, Wuhan Institute of Technology, Wuhan 430205, China

**Keywords:** zein, carvacrol, composite nanoparticles, sodium caseinate, pH-driven, antimicrobial activity

## Abstract

To reduce the application of synthetic additives in the field of food preservation, this study utilized carvacrol as an antibacterial agent, and zein and sodium caseinate as carriers, to prepare composite nanoparticles loaded with carvacrol by the pH-driven method. The composite nanoparticles of zein/sodium caseinate had an excellent encapsulation efficiency (77.96~82.19%) for carvacrol, and it had remarkable redispersibility. The results of Fourier transform infrared spectroscopy showed that the formation of the composite nanoparticles mainly depended on the hydrogen bond and the hydrophobic zone force, and thermal gravimetric analysis showed that carvacrol was loaded successfully into nanoparticles, and loading efficiency reached 24.9%. Scanning electron microscopy showed that the composite nanoparticles were spherical, with a particle size range of 50~200 nm, and through the free radical scavenging method and the plate counting method to confirm the particle has stronger antioxidant and antibacterial properties, and with the composite nanoparticles with poly (vinyl alcohol) film applied to the preservation of banana together, it was found that PVA film containing 5 wt% CA-loaded composite NPs can significantly extend the storage period of banana. Therefore, when the composite nanoparticles were applied to food packaging, they could effectively inhibit food spoilage and lengthen the shelf life of food, which displays potential application prospects in the food industry.

## 1. Introduction

In the food preservation industry, consumers are increasingly looking for healthier, greener options; therefore, it is necessary to seek natural and nontoxic antibacterial agents. Carvacrol (CA), also known as 5-isopropyl-2-methylphenol, is a natural compound extracted mainly from aromatic and medicinal plants [1], and it is generally recognized as a safe substance [2] by the U.S. Food and Drug Administration (FDA). On account of its remarkable antioxidant, antibacterial, antiviral, and other pharmacological effects [3,4], more and more researchers have paid attention to it in different applications. However, CA has some shortcomings, such as low water solubility [5], unique smell, and prone to oxidation, decomposition, or evaporation in air, light, or hot environments [6], which seriously restrict its application in the food preservation industry. In order to overcome these shortcomings, various routes have been developed to improve the stability and controlled-release properties of CA, including the preparation of liposomes [7], microcapsules, and emulsions [8], or directly adding CA essential oils (EOs) to films [9]. It is generally believed that reducing CA-loaded particles to nanometer scale can effectively improve their bioavailability, redispersity, and stability.

Zein is the main storage protein of corn, accounting for 45~50% of the total weight of corn protein, and it contains a large number of nonpolar amino acids, while it lacks basic amino acids and acidic amino acids [10]. Therefore, zein does not dissolve in pure water but is soluble in 60~90% (*v/v*) ethanol aqueous solution [11] and alkaline solution with a pH of 11.3~12.7 [12]. Generally, zein is a kind of biomolecular material with wide application prospects [13], as it is an abundantly renewable resource with excellent biodegradability, biocompatibility, hydrophobicity, and film-forming properties [14]. However, the nanoparticles (NPs) prepared by pure zein present some problems. On the one hand, as the isoelectric point of zein is close to neutral, zein NPs are not stable due to such weak electrostatic repulsion [15]. On the other hand, their hydrophobic outer surface results in very poor dispersion of NPs in water after drying, and therefore, they cannot be directly used in the food industry.

As a safe and harmless emulsifier with good surface activity, sodium caseinate (SC) was considered to effectively improve the redispersibility of zein NPs by combining with zein [16]. In addition, [17] found that SC and zein could be dissolved in a strong alkaline solution, and they would recombine with each other during the shifting process of acidic solution to neutral solution, forming coassembled binary zein/SC NPs, and the whole process can be realized by a pH-driven method. Under alkaline conditions, some bioactive compounds containing hydroxyl groups will undergo a deprotonation reaction [18], which can improve their water solubility. After neutralization, they can be protonated again and encapsulated into a biopolymer matrix resulting from the hydrophobic force and hydrogen bonding [19]. In other words, zein, SC, and CA can be codissolved in an alkaline solution, and then the ternary zein/SC/CA composite NPs can be prepared through pH value adjustment from alkaline to neutral. Compared with the traditional reverse solvent precipitation method [20], there are no organic solvents used in the pH-driven route to prepare composite NPs, demonstrating a much safer and more environmentally friendly method.

The main objective of this study is to prepare excellent capacity zein/SC/CA composite NPs by the pH-driven method, and make composite NPs have a smaller particle size, higher encapsulation efficiency, and better redispersibility. When applied to food packaging, zein/SC/CA nanoparticles were found to effectively improve the antioxidant properties and antibacterial properties of food, inhibit food spoilage, and greatly extend the shelf life of food, showing potential applications in the food industry.

## 2. Results

### 2.1. FT–IR Spectroscopy

**Figure 1:**
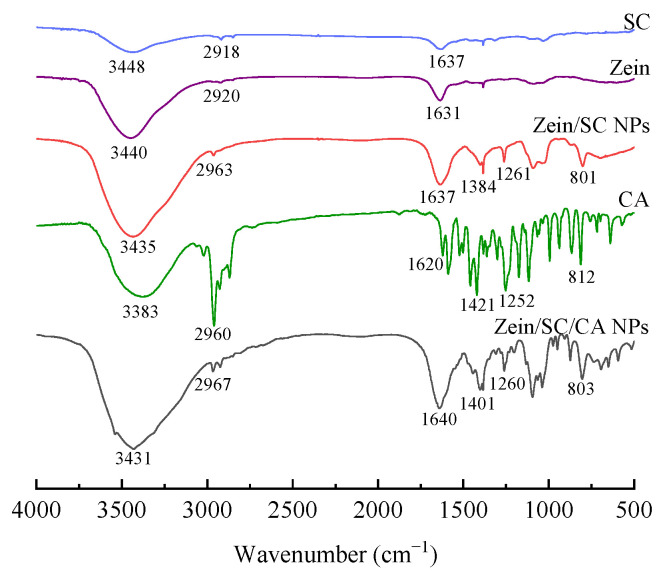
FT-IR image of SC, zein, zein/SC composite NPs, CA, and zein/SC/CA composite NPs.

The hydrogen bonds formation can be determined by FT–IR. In general, the higher the frequency of infrared absorption peaks, the stronger the hydrogen bond interaction between substances. FT–IR images of each sample and CA–loaded composite NPs are shown in Figure 1. Several characteristic peaks were observed in the spectra of SC and zein. The peaks at 3500~3200 cm^−1^ and 3000~2800 cm^−1^ represented the stretching of the hydrophilic O–H bond and the hydrophobic C–H bond, respectively, indicating a good amphiphilicity. In addition, peak distributions of the amide groups in the range of 1600~1400 cm^−1^ for the two proteins are similar [21]. For CA, due to the skeleton vibration of the benzene ring, there were four peaks in the region of 1650~1400 cm^−1^, and the peaks at 2960~2700 cm^−1^ were attributed to the alkyl C–H vibration. The peak around 2960 cm^−1^ was very strong and sharp, suggesting the surface-hydrophobic property of CA. For the binary zein/SC composite NPs, the spectra were quite similar to that of SC and zein, while there were two new peaks around 1261 cm^−1^ and 801 cm^−1^ resulting from the bending vibrations of C–O (ester group) and C–H (end group) in glucose-δ-lactone, respectively. For zein/SC/CA composite NPs, the spectra at 1450~800 cm^−1^ were significantly different from those of zein/SC composite NPs, and a vibration peak was observed at 1401 cm^−1^ attributed to the benzene ring vibration in CA, indicating that CA molecules were successfully embedded in the composite NPs. In addition, compared with CA, the peak around 2960 cm^−1^ was very weak in zein/SC/CA composite NPs, which may show the weak hydrophobicity in ternary composite NPs. Furthermore, the –OH peak was moved from 3440 cm^−1^ for the zein to 3435 cm^−1^ with the addition of SC, and it further shifted to a much lower wavenumber of 3431 cm^−1^ with CA loading. This big shifting of the –OH peak may indicate the strong hydrogen bonds formed among the amide bonds in zein, the amide bonds in SC, and the hydroxyl groups in CA [22]. From the above discussion, zein/SC/CA composite NPs demonstrated a stable structure due to the hydrogen bonding and the hydrophobic force.

### 2.2. EE of CA-Loaded Composite NPs

**Figure 2:**
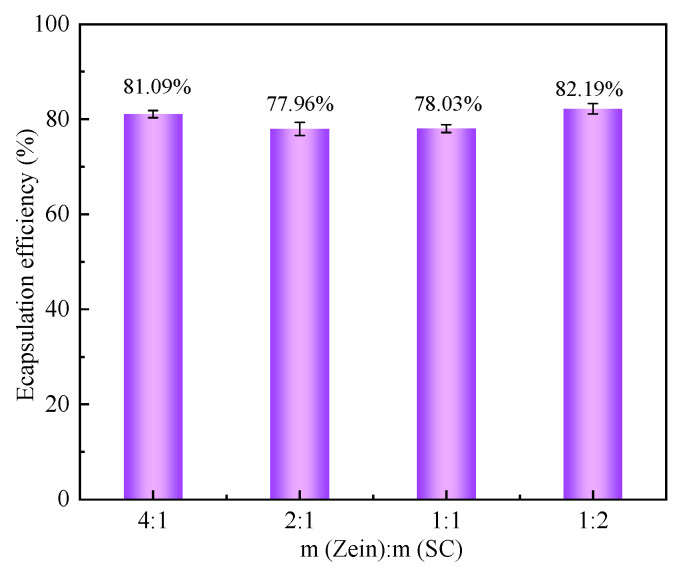
The EE of CA-loaded composite NPs with different zein/SC mass ratio (1:2, 1:1, 2:1, and 4:1).

EE refers to the percentage of the encapsulated substance (such as a drug) in the total amount of drug in liposome suspension. It is an important parameter that can reflect the degree of encapsulation of the compound by the carrier. Under a given CA mass, the EE of composite NPs was affected by the ratio of zein/SC mass. Figure 2 gives the effect of the zein/SC mass ratio on the EE of composite NPs. It can be found that the four zein/SC composite NPs all presented high EE values of around 80%. With the increase in SC mass, the EE value was gradually enhanced, and Wang et al. [23] also reported a similar phenomenon. The highest EE of composite NPs reached 82.19% with a zein/SC mass ratio of 1:2, which was about 8% larger than the maximum EE (74.2%) reported by Liu et al. [24]. Here, SC worked as an electrostatic stabilizer on the surface of zein/CA to prevent the aggregation of composite NPs, and thus the amount of SC is an important factor.

### 2.3. Analysis of Particle Size Distribution and Potential

**Figure 3:**
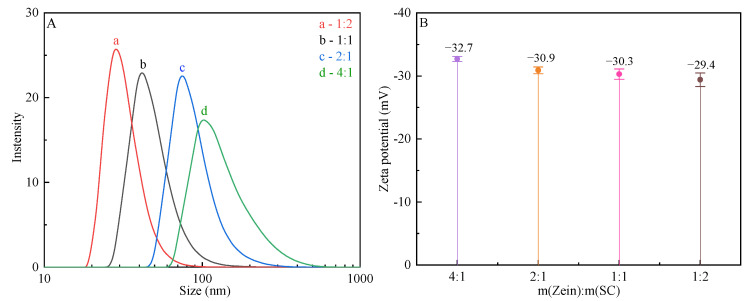
The effect on particle size distribution and potential of zein/SC composite NPs with different mass ratios (1:2, 1:1, 2:1, and 4:1), (**A**) particle size distribution, (**B**) zeta potential.

The effect of the zein/SC mass ratio on the particle size distribution and zeta-average potential is given in Figure 3. The particle size distribution of zein/SC/CA composite NPs at different mass ratios showed a single and relatively sharp peak (Figure 3A), indicating that the particle size distribution of composite NPs was very narrow. With the increase in SC content, the particle size gradually decreased. The average particle sizes of composite NPs were about 200 nm, 140 nm, 100 nm, and 70 nm for zein/SC mass ratios of 4:1, 2:1, 1:1, and 1:2, respectively. Hydrogen bonds were reported to be easily formed between zein and SC molecules [25], and the density of hydrogen bonds between zein and SC could be large with high SC content, resulting in a reduced particle size and an improved dispersion of composite NPs.

In Figure 3B, it can be found that the effect of zein/SC mass ratios on the zeta potential of composite NPs displayed a similar trend to that in particle size distribution. The zeta-average potential was −32.7 mV for m (zein)/m (SC) = 4:1, and it was −29.4 mV for m (zein)/m (SC) = 1:2. Generally speaking, the surface net charge of composite NPs gradually increased with high content of SC. However, it is interesting that there is an inverse appearance. As the particle size greatly decreased, the specific surface area of composite NPs became larger. As a result, the surface charge density of composite NPs dropped with smaller particle sizes.

### 2.4. Analysis of Redispersibility

**Figure 4:**
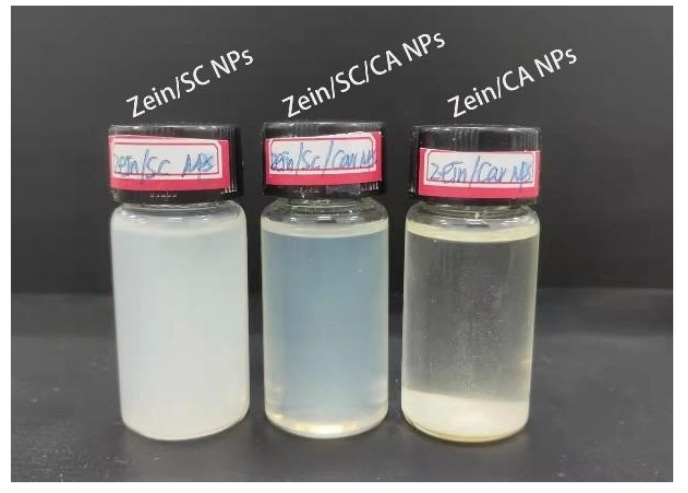
The redispersibility of zein/SC, zein/CA, and zein/SC/CA composite NPs.

The freeze-dried reagent can not only simplify the transport mode but also improve the stability of the reagent. Redispersibility is an important index for the performance of lyophilized powder. Figure 4 shows the dissolved states of zein/SC, zein/CA, and zein/SC/CA composite NPs in deionized water with the same concentration. It can be seen from Figure 4 that the zein/CA composite NPs dispersion without SC showed an obvious stratification after standing for a period. Zein/CA composite NPs in the dispersion precipitated rapidly at the bottom of the bottle, and the upper aqueous solution was still clear and transparent. However, composite NPs added with SC showed a good redispersibility and could disperse in water to form a homogeneous solution, which was consistent with the report of Patel [26]. In addition, the zein/SC composite NPs dispersions appeared milky white, while the zein/SC/CA composite NPs dispersions were light blue, and such difference was possibly due to the combination of CA with the nonpolar amino acids of zein to form smaller NP dispersion.

### 2.5. Storage Stability of Composite NPs

**Figure 5:**
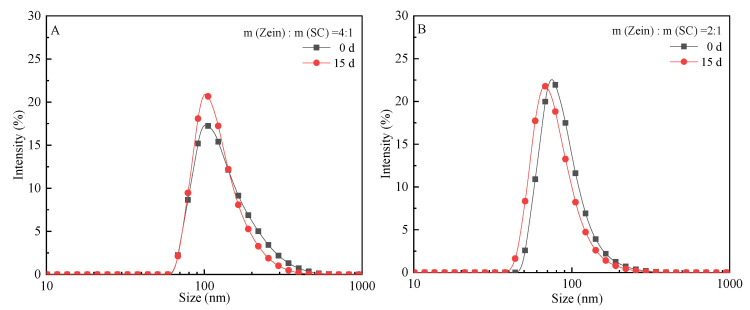

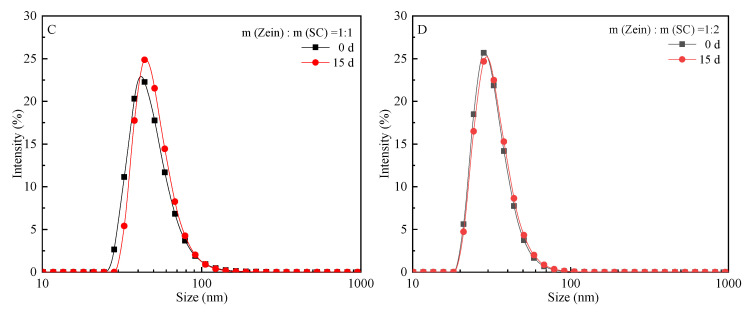
The size of zein/SC/CA composite NPs with different mass ratios, after storage for 0 and 15 days. (**A**) zein/SC mass ratios of 4:1; (**B**) zein/SC mass ratios of 2:1; (**C**) zein/SC mass ratios of 1:1; (**D**) zein/SC mass ratios of 1:2.

Figure 5 shows the changes in the particle size of CA-loaded composite NPs with different zein/SC mass ratios when stored for 0 d and 15 d, respectively. Zein/SC/CA composite NPs prepared by the pH-driven method were soluble in water, but their storage stability was quite different. As shown in Figure 5A,B, the average particle size decreased after 15-day storage for composite NPs with zein/SC mass ratios of 4:1 and 2:1, and the particle size distribution was more concentrated. Such a phenomenon was mainly attributed to the agglomeration of unstable composite NPs into large particles at the bottom of the bottle. In Figure 5C, the particle size distribution was more concentrated after 15 days for composite NPs in the dispersion with the mass ratio of 1:1, but the average particle size increased due to the aggregation of some composite NPs. However, when the zein/SC mass ratio is 1:2, the particle size and distribution of the dispersion have nearly no change after 15 days (Figure 5D). The results indicated the storage stability of composite NPs depended on the zein/SC mass ratio, and the optimized ratio is 1:2.

Table 1 lists the average potential and PDI value of CA-loaded composite NPs with different mass ratios of zein/SC when stored for 0 and 15 days. The zeta potential increased after 15-day storage for composite NPs with the mass ratios of zein/SC were 4:1 and 2:1, while it decreased for composite NPs with the mass ratios of zein/SC were 1:1 and 1:2. The potential value changed the most for the mass ratio of zein/SC was 1:2, showing a fall from −29 mV to −37 mV. Such a phenomenon was mainly attributed to more carboxylate being exposed on the outer surface of the particles after storage. PDI value was usually used to characterize the particle size distribution of suspension, and a lower PDI indicated a more uniform particle size distribution. By comparing the changes in PDI values of the dispersions at 0 d and 15 d, it can be found that the PDI values of the four groups of dispersions decreased to different degrees after 15 days, indicating the better distribution uniformity of composite NPs in the dispersions during the standing. In short, the composite NPs with the mass ratio of zein/SC was 1:2, the storage stability was the best. The particle size had nearly no change during the standing, and the stability was better as well.

### 2.6. Particle Structures Studied with FE–SEM

**Figure 6:**
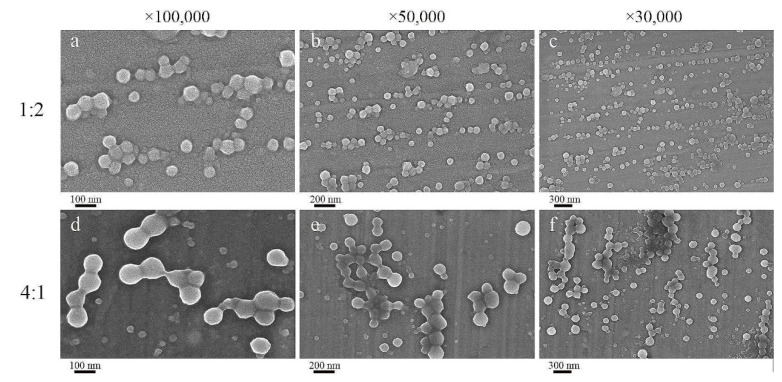
FE-SEM images of CA-loaded composite NPs: m (zein)/m (SC) = 1:2 ((**a**) 100 nm; (**b**) 200 nm; (**c**) 300 nm); m (zein)/m (SC) = 4:1 ((**d**)100 nm; (**e**) 200 nm; (**f**) 300 nm).

The morphology of composite NPs was observed by FE-SEM. As shown in Figure 6, the CA-loaded composite NPs had a perfect spherical structure. The particle size of composite NPs with a zein/SC mass ratio of 1:2 was about 50~90 nm (Figure 6a–c), and the distribution was relatively uniform. A few composite NPs were smaller than 50 nm, which were hollow particles formed by zein self-assembly. In addition, there were a small number of composite NPs with a particle size of about 200 nm, resulting from the agglomeration of composite NPs due to the lack of charge and repulsion on the surface. The particle size of composite NPs with a zein/SC mass ratio of 4:1 was about 90~250 nm (Figure 6d–f). Moreover, the particles adhered to each other and agglomerated together, indicating that a small amount of SC could not make the nanoparticles stable. The results show that a proper amount of SC could interact with the protein to form the particles of negatively charged COO^-^ on the surface, which made the particles repel each other and hence improved their stability. However, the composite nanoparticles without SC possessed less surface charge, which led to their instability through the formation of large particles.

### 2.7. Analysis of TG

**Figure 7:**
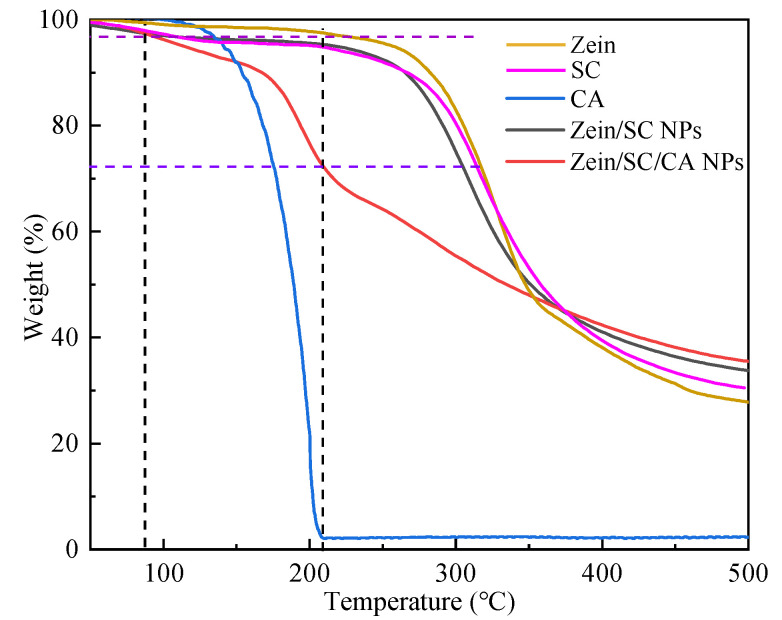
TGA pattern of zein, SC, CA, zein/SC composite NPs, and zein/SC/CA composite NPs.

TGA was used to further study the thermal stability of the samples and the LE of CA-loaded composite NPs. Figure 7 shows the TGA pattern for zein, SC, CA, zein/SC and zein/SC/CA composite NPs. The mass loss of zein and SC could be divided into two stages. The first stage was from 50 °C to 210 °C, and the mass loss was 2.57% and 5.22% for zein and SC, respectively, mainly resulting from the evaporation of water in the sample. The second stage was from 210 °C to 400 °C, the mass loss of zein was 59.14%, and that of SC was 57.75%, which were mainly attributed to the breakdown of peptide bonds and amino acids in the protein structure [27]. When the temperature was 500 °C, the residual mass of zein was only 27.49%, and that of SC was 30.29%, and they mainly included hydrocarbons that were difficult to decompose in the nitrogen atmosphere [28]. When the temperature rose to 210 °C, CA would be volatilized completely. Zein/SC composite NPs were taken as a control, and about 24.9% mass loss was regarded as CA LE in the zein/SC/CA composite NPs. Therefore, taking zein/SC composite NPs as the control at 210 °C, the relative mass loss of zein/SC/CA composite NPs was about 24.9%, which was the LE of CA in composite NPs.

### 2.8. Analysis of Antioxidant Capacity

**Figure 8:**
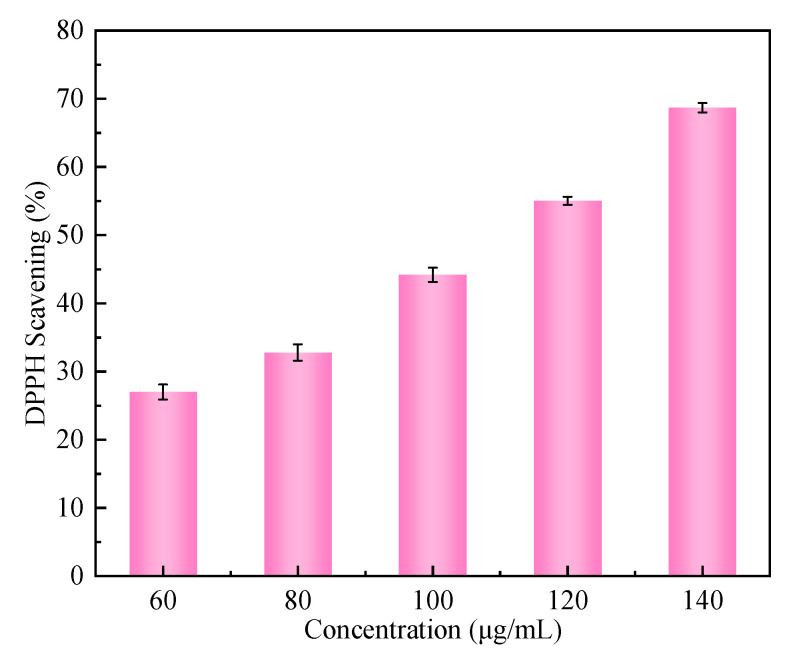
DPPH scavenging rate of zein/SC/CA composite NPs with different mass concentrations (60, 80, 100, 120, and 140 μg/mL).

In vitro, antioxidant activities of some plant EOs had been reported everywhere, and such activities were mainly attributed to the content of phenolic components, especially thymol and CA [29]. Here, the free radical scavenging activity of CA-loaded composite NPs was investigated. In Figure 8, the antioxidant capacity of CA-loaded composite NPs increased from 27% to 68.69%. With the increase in the mass concentration of composite NPs from 60 to 140 μg/mL, the DPPH clearance rate obviously increased during the whole process, indicating that the higher mass concentration was beneficial to the antioxidant capacity.

### 2.9. Analysis of Antimicrobial Activity

**Figure 9:**
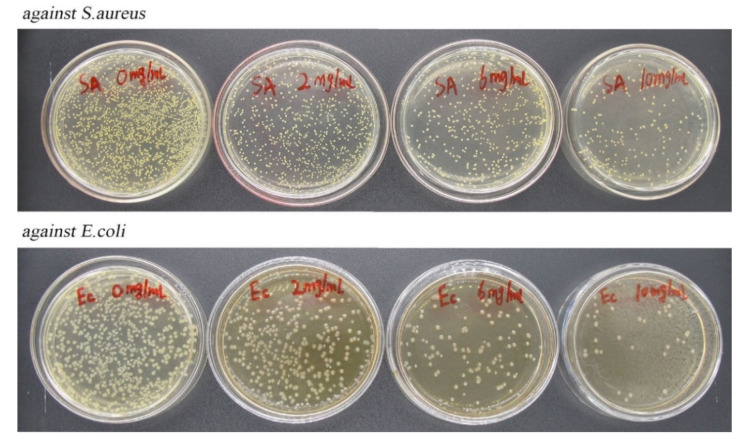
Antibacterial effect of zein/SC/CA composite NPs with different mass concentrations (0, 2, 6, and 10 mg/mL) against *S. aureus* and *E. coli.*

In Burt’s [30] study, it was found that carvacrol had significant inhibitory effects on Staphylococcus aureus and Escherichia coli. Therefore, the plate counting method was used to study the antibacterial activity of composite NPs. The antibacterial effects of CA-loaded composite NPs against *S. aureus* and *E. coli* were shown in Figure 9. With the increase in mass concentration, the number of bacterial colonies on the plate decreased gradually, indicating that CA-loaded composite NPs presented a good antibacterial capability against *S. aureus* and *E. coli*. When the mass concentration of composite NPs was 0, 2, 6, and 10 mg/mL, the antibacterial rates of *S. aureus* reached 0%, 69%, 76%, and 91%, respectively, and the antibacterial rates of *E. coli* reached 54%, 72%, and 85%, respectively. The results indicated that the CA-loaded composite NPs had a potential antibacterial effect against *S. aureus* and *E. coli*.

### 2.10. CA-Loaded Composite NPs in Food Storage

**Figure 10:**
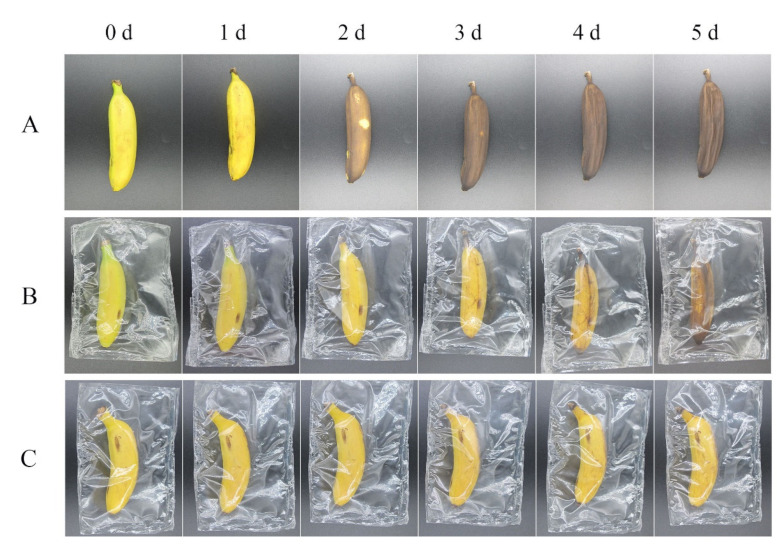
CA-loaded composite NPs for banana storage. ((**A**) storage in air; (**B**) storage in PVA film; (**C**) storage in PVA film containing 5 wt% CA-loaded composite NPs).

To evaluate the preservation capability of CA-loaded composite NPs to keep the freshness of food, PVA films containing CA-loaded composite NPs were prepared, and fresh bananas were thus wrapped by these protective films and then kept at 30 °C (relative humidity: 50%). Figure 10 gives the results of CA-loaded composite NPs for banana storage. Sample A was the bare banana without any wrapping films, Sample B was the banana wrapped with pure PVA films, while Sample C was the banana wrapped with PVA containing CA-loaded composite NPs. As seen in Figure 10, the banana surfaces of the three groups were originally smooth and yellow (0 days). After 2 days, the surface showed obvious color change from yellow to brown for Sample A without any wrapping, while they still kept yellow for Samples B and C. Four days later, some wrinkles and completely brown color can be seen on the surface of Sample A. For Sample B wrapped with PVA film, the surface showed very little brown color after 3-day storage; however, clear color change appeared after 5 days. For Sample C wrapped with PVA film containing 5 wt% CA-loaded composite NPs, the surface kept its yellow through the whole 5-day storage, and there was nearly no color change during the experiments. These phenomena indicated that CA-loaded composite NPs can effectively maintain the color and appearance of banana in combination with PVA film. Therefore, CA-loaded composite NPs exhibited great potential for food preservation.

## 3. Discussion

Most of the active ingredients extracted from plants have good antioxidant properties and broad-spectrum antibacterial properties [31], which can be used as natural preservatives in the food preservation industry. However, the lipid-soluble active ingredients have some problems such as insoluble or insoluble in water, unstable in air, and pungent odor. In order to make better use of natural preservatives, researchers in recent years have mainly used encapsulation technology [32] to improve the utilization rate of bioactive components and their solubility and stability in aqueous media. Nanotechnology in improving the existing technology and in the new scientific development has many prospects [33], especially nanoencapsulation technology, which can be effective to solve the problems existing in the active ingredient, when composite NPs as carrier can effectively cover up their own bad smell, in the subsequent application of nanoscale size were also more likely to spread and dispersed more evenly. In this study, we prepared a kind of composite NPs with antibacterial and antioxidant properties by using zein as encapsulation carrier, CA as antibacterial agent, and SC as stabilizing agent.

In this study, zein/SC/CA composite NPs were prepared by the pH-driven method, and the FT-IR results showed that the absorption peaks of the samples were significantly different, which confirmed that hydrogen bond and hydrophobic force were the main forces for the formation of composite NPs. The composite NPs were characterized by SEM and DLS. The particle size ranged from 50 nm to 200 nm, and the particles were spherical. TGA results show that the composite NPs have good thermal stability, and the loading rate of CA reaches 24.9%. By dispersing the freeze-dried composite NPs in water and the DPPH radical scavenging method, the composite NPs have good redispersibility and antioxidant properties. The antibacterial test results of the composite NPs on *S. aureus* and *E. coli* show that the composite NPs have excellent antibacterial properties. In practice, bananas in PVA films containing 5 wt% composite NPs were the freshest, which indicates that composite NPs have great potential in the field of food preservation.

Compared with our published studies [34], the composite NPs prepared in this study have better structure and performance. Published studies have used the antisolvent precipitation method to prepare CA-loaded composite NPs. The average particle size of composite NPs was about 130 nm, and the LE and free radical scavenging rates were 18% and 54%, respectively. The inhibition rates of the composite NPs against *S. aureus* and *E. coli* were 80% and 66.7%, respectively. In practice, the color of bananas stored in the PVA film containing 5 wt% CA-loaded composite NPs had changed at 23 °C for 4 days. Under the same mass fraction of CA, the average particle size of the composite NPs prepared in this study is about 70 nm, which is reduced by 50 nm compared with the published study. The LE rate and free radical scavenging rate are increased by 6.9% and 16% compared with the published study, respectively. The inhibition rates of *S. aureus* and *E. coli* were increased by 11% and 18.3%, respectively. In this study, the surface of bananas in PVA film containing 5 wt% CA-loaded composite NPs hardly changed when stored at 30 °C for 5 days

## 4. Materials and Methods

### 4.1. Materials

Zein was provided by Beijing Solarbio^®^ Science & Technology, Co. Ltd. (purity ≥ 95%, Beijing, China). CA was purchased from Marklin (purity ≥ 99%, Shanghai, China). SC powder with a protein content greater than 99% (*w/w*) was provided by Shanghai Tixiai Chemical Industry Development Co., Ltd. (Shanghai, China). The other chemicals were purchased from Sinopharm Chemical Reagent Co., Ltd. (analytical grade, Shanghai, China). All solutions were prepared with ultrapure water.

### 4.2. Preparation of CA-Loaded Composite NPs

First, 25 mL NaOH (3 M) solution and 2.5 g CA were added to a round-bottom flask at the same time. After full mixing, they were heated in a 120 °C silicone oil bath for 10 min to obtain a transparent deprotonated CA alkali solution. Later, 1 mL deprotonated CA alkali solution was completely mixed with 19 mL deionized water. Finally, 0.1 g zein was dissolved in the above solution and stirred at 800 rpm for 30 min until no particles were visible.

Different masses of SC (0.025, 0.05, 0.1, and 0.2 g) were separately dissolved in the above zein/CA aqueous solution with the corresponding zein/SC ratios of 4:1, 2:1, 1:1, and 1:2, respectively, and stirred at 800 rpm in a magnetic mixer for 40 min. Finally, 20% (*w/w*) glucose-δ-lactone (GDL) was added to the zein/SC/CA alkaline mixture to adjust the pH to 7.0. All composite NPs were freeze-dried at −48 °C for 2 days (Alpha 1-4 LD Plus, Christ, Germany). The freeze-dried composite NPs were ground into powder for later use.

### 4.3. Analysis of Fourier Transform Infrared (FT–IR) Spectroscopy

The sample was mixed with dried KBr powder at a mass ratio of 1:100 and pressed into circular pieces. Infrared spectrograms of samples were measured using an FT–IR spectrometer (Nicolet 6700, Thermo Fisher Scientific Inc., Waltham, MA, USA), and spectrograms were analyzed using the OMNIC 8.2 software package (Thermo Fisher Scientific Inc., Waltham, MA, USA). Samples test conditions: the scanning range of 400~4000 cm^−1^, resolution of 4 cm^−1^, taking 32 scans.

### 4.4. Encapsulation Efficiency (EE) of CA-Loaded Composite NPs

According to Wang’s method [35] with slight modification, the standard curve of CA (Y = 0.01439 X + 0.0061, R^2^ = 0.9996) was established. CA was dissolved in ethanol to prepare standard solutions (10 µg/mL~50 µg/mL). The absorbance of the solution at 276 nm was determined by a UV spectrophotometer (Lambda 35, Perkin Elmer, Waltham, MA, USA); ethanol was used as a blank control group. Then, the standard curve equation of CA concentration (X)—absorbance value (Y) was fitted.

Afterward, 4 mL of freshly prepared composite NPs dispersion was mixed with 16 mL petroleum ether, and the mixture was vortically oscillated for 10 min. Then, 0.5 mL organic phase was taken and placed in the fume hood for 30 min to make the petroleum ether completely volatilized. Subsequently, 4 mL of anhydrous ethanol was added to the reagent flask. The absorbance of the solution at 276 nm was determined by a UV spectrophotometer (Lambda35, Perkin Elmer). The EE was then calculated according to (1):

(1) $$\mathrm{EE}\left(\%\right)=\frac{\lambda_{1}–\lambda_{2}}{\lambda_{1}}\times 100\%$$

where λ_1_ represents the total mass of CA, and λ_2_ represents the free mass of CA in the dispersion.

### 4.5. Analysis of Particle Size Distribution and Potential

At 25 °C, all samples were diluted with ultrapure water to an appropriate concentration, and the particle size distribution and average potential of NPs in the dispersion were measured by a laser particle size analyzer (Zetasizer Nano-ZS90, Malvern, UK). Each sample was measured in triplicate, and the data were averaged.

### 4.6. Redispersibility Evaluation

The freeze-dried zein/SC, zein /CA and zein/SC/CA composite NPs were dissolved in water and prepared into dispersions with a concentration of 2 mg/mL. After standing for 3 h, the redispersibility of nanoparticles in three groups of dispersions was observed.

### 4.7. Storage Stability Evaluation

The storage stability of composite NPs was measured by dynamic light scattering (DLS). Briefly, freeze-dried composite NPs with different zein/SC ratios (1:2, 1:1, 2:1, and 4:1) were prepared into dispersions of 2 mg/mL. The changes of particle size distribution, average potential and polydispersity index (PDI) in dispersants stored for 0 and 15 days were observed at 25 °C. Each sample was measured in triplicate, and the data were averaged.

### 4.8. Analysis of Geometric Morphology

Sprinkle the freeze-dried powder evenly on the conductive plate and then spray gold. FE-SEM (Zeiss SIGMA 300, Oberkochen, Germany) at 3 kV was used to characterize the microstructure of CA-loaded composite NPs.

### 4.9. Thermal Gravimetric Analysis (TGA)

The loading efficiency (LE) of CA-loaded composite NPs was characterized by TGA-5500 (TA instruments, Peoria, IL, USA). About 5 mg samples were heated from room temperature to 500 °C at a rate of 10 °C/min with a N_2_ flow rate of 20 mL/min. The weight loss was analyzed using Trios 5.1 software. When the CA mass was reduced to 0 mg, the mass difference between the total weight loss of zein/SC NPs and zein/SC/CA composite NPs was used as LE.

### 4.10. Antioxidant Activity Evaluation

The antioxidant activity of CA-loaded composite NPs was determined by the 1,1-diphenyl-2-picrylhydrazyl (DPPH) radical method [36]. DPPH scavenging rate can reflect the antioxidant activity of composite NPs. Add an equal volume of DPPH ethanol solution (40 mg/mL) and CA-loaded composite NPs dispersion of different mass concentrations (0, 60, 80, 100, 120, and 140 µg/mL) to the brown reagent bottle. After mixing evenly, leave to stand at 25 °C in the dark for 1 h. With DPPH ethanol solution as the blank control, the absorbance value of the mixed solution at 525 nm was measured with a UV spectrophotometer (Lambda 35, Perkin Elmer). The DPPH scavenging rate was then calculated according to (2):

(2) $$\mathrm{DPPH}\;\mathrm{scavenging}\left(\%\right)=\frac{A_{0}–A_{1}}{A_{0}}\times 100\%$$

where A_0_ represents the absorbance of the DPPH ethanol solution, and A_1_ represents the absorbance of the test sample.

### 4.11. Antimicrobial Activity Evaluation

The plate counting method was used to investigate the antimicrobial activity of CA-loaded composite NPs on *Staphylococcus aureus* (*S. aureus*) and *Escherichia coli* (*E. coli*). Briefly, the CA-loaded composite NPs were dissolved in normal saline to prepare dispersions of different mass concentrations (0, 2, 6, and 10 mg/mL, respectively) and irradiated by a UV lamp for 1 h to achieve aseptic conditions. *S. aureus* and *E. coli* were inoculated into solid medium by the plate scribing method and cultured at 37 °C for 1 day to obtain purified strains. Single colonies of *S. aureus* and *E. coli* were added into the two groups of 15 mL liquid medium. After shaking for 24 h, the concentration of the bacterial suspension was diluted to 10^8^ CFU/mL. Then, 8.9 mL liquid medium and 1.0 mL CA-loaded composite NPs dispersion were evenly mixed, and 0.1 mL diluted bacterial suspension was added to the mixture. After the mixture was evenly mixed, the culture was continued for 1 day at 37 °C. Finally, the bacterial solution was diluted to an appropriate concentration, 0.4 mL was taken and spread on solid medium for further culture for 24 h, and the number of colonies was recorded. Composite NPs without CA were used as a blank control group, and all steps were performed in a sterile environment. The antibacterial activity (I) was then calculated according to (3):

(3) $$I=\frac{I_{0}–I_{1}}{I_{0}}\times 100\%$$

where I_0_ is the number of bacterial colonies in the blank control, and I_1_ is the number of bacterial colonies in test samples.

### 4.12. CA-Loaded Composite NPs in Food Storage

Fresh bananas treated in different ways were divided into three groups and stored at 30 °C (relative humidity: 50%) for 5 days. The daily changes of the three groups were recorded by photos. Unpacked fresh banana was used as a blank control. The banana wrapped in a bag prepared with PVA film was the control group, and the banana wrapped in a bag prepared with PVA film containing CA-loaded composite NPs was the experimental group.

## 5. Conclusions

In this study, the pH-driven method was used to prepare the composite NPs with CA loading. SC was added to the surface of the composite NPs to improve the storage stability and redispersibility so that the antibacterial and antioxidant properties of carvacrol could be better improved. The results of FT–IR and TGA showed that CA was loaded successfully in the composite NPs, and the formation of the composite NPs was mainly attributed to hydrogen bonds between the three substances. With a zein/SC mass ratio of 1:2, the composite NPs exhibited small particle size, high encapsulation rate, good redispersibility, and storage stability, and they showed excellent antioxidant and antibacterial properties. Zein/SC/CA composite NPs have demonstrated promising applications in food preservation and shelf-life extension.

## Figures and Tables

**Table 1:** The CA-loaded composite NPs with different mass ratios of zein/SC of average potential and PDI values, after storage for 0 and 15 days.

Sample	0 d		15 d	
	Potential (mV)	PDI	Potential (mV)	PDI
4:1	−32.7 ± 0.404	0.176 ± 0.012	−28.87 ± 1.159	0.125 ± 0.0230
2:1	−30.9 ± 0.529	0.155 ± 0.005	−29.78 ± 1.210	0.139 ± 0.0093
1:1	−30.3 ± 0.814	0.195 ± 0.007	−31.90 ± 2.615	0.172 ± 0.0027
1:2	−29.4 ± 1.069	0.358 ± 0.004	−37.27 ± 2.250	0.289 ± 0.0197

Note: Values are expressed as mean ± standard deviation (n = 3).

## Data Availability

Not applicable.
